# Muscle synergy-informed neuromusculoskeletal modelling to estimate knee contact forces in children with cerebral palsy

**DOI:** 10.1007/s10237-024-01825-7

**Published:** 2024-03-09

**Authors:** Mohammad Fazle Rabbi, Giorgio Davico, David G. Lloyd, Christopher P. Carty, Laura E. Diamond, Claudio Pizzolato

**Affiliations:** 1 Griffith Centre of Biomedical and Rehabilitation Engineering (GCORE), Gold Coast, and Advanced Design and Prototyping Technologies Institute, Gold Coast, QLD 4222 Australia; 2 School of Health Sciences and Social Work, Griffith University, Gold Coast, QLD 4222 Australia; 3 Department of Industrial Engineering, Alma Mater Studiorum, University of Bologna, 40136 Bologna, Italy; 4 Medical Technology Lab, IRCCS Istituto Ortopedico Rizzoli, Bologna, Italy; 5 Department of Orthopaedic Surgery, Children’s Health Queensland Hospital and Health Service, Brisbane, QLD 4101 Australia

**Keywords:** Muscle synergy, EMG-driven, NMSK modelling, Joint contact force, Knee loading, Gait

## Abstract

Cerebral palsy (CP) includes a group of neurological conditions caused by damage to the developing brain, resulting in maladaptive alterations of muscle coordination and movement. Estimates of joint moments and contact forces during locomotion are important to establish the trajectory of disease progression and plan appropriate surgical interventions in children with CP. Joint moments and contact forces can be estimated using electromyogram (EMG)-informed neuromusculoskeletal models, but a reduced number of EMG sensors would facilitate translation of these computational methods to clinics. This study developed and evaluated a muscle synergy-informed neuromusculoskeletal modelling approach using EMG recordings from three to four muscles to estimate joint moments and knee contact forces of children with CP and typically developing (TD) children during walking. Using only three to four experimental EMG sensors attached to a single leg and leveraging an EMG database of walking data of TD children, the synergy-informed approach estimated total knee contact forces comparable to those estimated by EMG-assisted approaches that used 13 EMG sensors (children with CP, *n* = 3, *R*^2^ = 0.95 ± 0.01, RMSE = 0.40 ± 0.14 BW; TD controls, *n* = 3, *R*^2^ = 0.93 ± 0.07, RMSE = 0.19 ± 0.05 BW). The proposed synergy-informed neuromusculoskeletal modelling approach could enable rapid evaluation of joint biomechanics in children with unimpaired and impaired motor control within a clinical environment.

**Supplementary Information:**

The online version contains supplementary material available at 10.1007/s10237-024-01825-7.

## Introduction

Cerebral palsy (CP) is a lifelong neurological disorder caused by a brain injury that occurred during birth or in the neonatal period and is characterised by alterations of movement and postural control (Dodd et al. 2002). Aberrant muscle activity in individuals with CP is likely caused by abnormal motor control, a reduced number of motor units, and increased excitability of alpha and gamma motor neurons (Mockford and Caulton 2010; Bar-On et al. 2015). These neural and non-neural impairments manifest in altered gait biomechanics (Wren et al. 2005). To understand muscle contributions to impaired gait biomechanics, muscle activity profiles from a small number of lower limb muscles are commonly incorporated into clinical decision-making algorithms (Miller et al. 1997). However, these data alone are not sufficient to understand the altered muscle forces and joint contact forces in children with CP (Steele et al. 2012; Davico 2019), which are a plausible cause of the maladaptive processes associated with bone deformations (Bosmans et al. 2014; Carriero et al. 2014; Kainz et al. 2021). The ability to monitor these internal biomechanical factors may contribute to improved understanding of the progression of bone deformities observed in paediatric population with CP and help establishing appropriate surgical interventions (Modlesky and Zhang 2020). Nonetheless, measurement of muscle and joint contact forces are invasive and therefore not suitable in a clinical setting.

Computational neuromusculoskeletal (NMSK) modelling is a non-invasive approach to estimate joint contact forces. Within NMSK modelling, several methods currently exist to compute the activity of muscles during movement, which combined with appropriate musculoskeletal geometry and contact models, enable estimation of joint contact forces. For example, static optimisation estimates muscle activity by minimising a target cost function (e.g. sum of squared muscle activations) while satisfying the dynamics of motion (Anderson and Pandy 2001; Shuman et al. 2019b). In this approach, muscle excitations estimated by the model reflect neither physiological muscle activity (Veerkamp et al. 2019; Davico et al. 2020) nor muscle co-contractions (Lloyd and Besier 2003). In contrast, electromyogram (EMG)-driven methods require a set of experimental EMG recordings to estimate muscle forces and in turn predict plausible joint moments (Lloyd and Besier 2003). Most recent EMG-assisted approaches combine experimental EMG data with static optimisation to synthetise unmeasured muscle excitations and track external joint moments (Sartori et al. 2014; Pizzolato et al. 2015). While static optimisation requires no measured EMG data, the latter are necessary for any EMG-informed method (e.g. EMG-assisted or EMG-driven), which have consistently demonstrated excellent ability to estimate measured joint contact forces (Gerus et al. 2013; Saxby et al. 2016; Hoang et al. 2018, 2019; Bennett et al. 2022). However, the use of EMG-informed approaches in clinical settings has been limited by practical challenges in collecting EMG data from a sufficient number of muscles due to time constraints and difficulties in applying electrodes, as well as encumbrance to gait, especially in smaller children. Hence, only four to five EMGs are commonly recorded from children with CP during a gait assessment while the rest of the muscles remain unmeasured (Steele et al. 2015). Given that EMG data from up to 16 muscles may be required to develop a comprehensive EMG-informed NMSK model (Sartori et al. 2013; Ao et al. 2020), new methods that employ only a few experimental EMG recordings to estimate joint moments and contact forces would facilitate significant advances in the clinical assessment of these cohorts.

Estimation of unmeasured muscle activity may be facilitated using muscle synergies, which refer to the coordinated activation of a group of muscles during any rhythmic task (e.g. walking) (Ferrante et al. 2016). Muscle synergies are mathematically extracted from processed EMG data (i.e. muscle excitations), which results in two matrices known as the (i) excitation primitives, and (ii) muscle synergy weights (Cheung et al. 2005). Excitation primitives represent the magnitude-timing profile of the synergy, while muscle synergy weights represent the magnitude of each muscle’s excitation projected onto each excitation primitive (Lambert-Shirzad and Van der Loos 2017). Linear combinations of excitation primitives and muscle synergy weights can be used to reconstruct the original muscle excitations with errors, depending on number of synergies extracted. For instance, for walking, three to five muscle synergies can account for at least 90% variance of the original muscle excitations in healthy children (Rozumalski et al. 2017), while in children with CP, a smaller number of synergies can achieve the same level of variance (Steele et al. 2015). The evidence of simplified control strategies employed by individuals with CP led us to speculate that synergies from children with CP could be considered a subset of those used by typically developing (TD) children. This speculation was supported by the ability to reconstruct a full set of lower limb muscle excitations for children with CP combining minimal experimental EMG data and an existing database of muscle excitations from TD children (Rabbi et al. 2022).

Albeit muscle synergy extrapolation methods are just emerging and are not extensively validated, their integration with NMSK models could enable investigating internal biomechanics using minimal experimental EMG data. Earlier studies incorporated muscle synergy approaches into the development of NMSK models in healthy adults (Allen and Neptune 2012), stroke survivors (Allen et al. 2013; Meyer et al. 2016), and children with CP (Shuman et al. 2019a, b) to estimate joint moments and unmeasured muscle excitations. Those methods focused on tracking synergy excitation primitives as part of the optimisation, resulting in similar EMG tracking performance compared to an EMG-driven approach (Allen and Neptune 2012; Sartori et al. 2012; Walter et al. 2014). A NMSK model of a healthy and a post-stroke participant were also developed, wherein a calibrated EMG-driven approach was used to estimate unmeasured muscle excitations and hip joint moments (Ao et al. 2020). Nevertheless, all these studies extracted muscle synergies using a full set of measured EMG data, which is not desirable in clinical settings.

This study aimed to develop a synergy–informed NMSK modelling workflow that combined synergy extrapolation (Rabbi et al. 2022) with EMG-informed modelling (Pizzolato et al. 2015). We explored whether this approach, when using a small number of experimental EMG recordings, could produce plausible estimates of lower limb joint moments and knee contact forces in a TD paediatric population and in children with CP, during walking.

## Methods

The next subsections of the methods provide a summary of the study participants characteristics (2.1 Participants), followed by a brief description of how experimental data were processed (2.2. Data processing). Processed data were used to scale the musculoskeletal model geometry to each individual, followed by initial tuning of musculotendon unit (MTU) parameters and calculation of MTU kinematics and joint moments (2.3. NMSK model scaling and parameter tuning). Musculoskeletal data were then combined with experimental muscle excitations and used to first calibrate all the model’s parameters and then estimate muscle forces and a complete set of muscle excitations via EMG-assisted and static optimisation approaches (2.4 NMSK model calibration and execution). The complete set of muscle excitations from TD children, generated via the EMG-assisted method, was used as database for the synergy extrapolation method. A full set of muscle excitations, extracted via synergy extrapolation and a low number of experimental muscle excitations from children with CP, were then used as input to the calibrated NMSK model to calculate muscle forces (2.5 Synergy-informed NMSK modelling). Muscle forces calculated with each of the modelling approaches were input to a contact model (2.6 Knee joint contact model) to calculate knee contact forces. Contact forces calculated using the proposed synergy-informed workflow were compared to those calculated from EMG-assisted and static optimisation to assess performance (2.7 Comparing synergy-informed, EMG-informed, and static optimisation NMSK modelling predictions), with information criteria used to evaluate the complexity of the different modelling solutions (2.8 Information criteria applied to NMSK modelling). Finally, performed statistical analyses were described (2.9 Statistical analyses).

### Participants

Clinical gait data from a previous study (Davico et al. 2022) were used for analysis. Data were collected on three children with CP and three age-matched TD children (Table 1). All children with CP were independent walkers, i.e. classified as I (*n* = 2) or II (*n* = 1) according to the gross motor function classification scale (GMFCS). Participants with CP were excluded if they had received musculoskeletal surgery (e.g. muscle lengthening or botulinum injection) in the six months prior to the testing. Thirteen wireless bipolar EMG sensors (Zerowire, Aurion, Milan, IT. 1000 Hz) were placed on selected muscles (Table 2) of the right (TD participants) or most affected lower limb (participants with CP) by an experienced physiotherapist. Trajectories of 51 markers were collected using a 10-camera motion capture system (Vicon Motion System, Oxford, UK; 100 Hz) while the subjects performed overground walking trials at preferred walking speed (i.e. 0.9 ± 0.1 m/s). Motion capture and EMG data were recorded for 14 gait cycles (i.e. between consecutive heel-strike of the instrumented leg). The study was approved by the Children’s Health Queensland Hospital and Health Services Human Research Ethics Committee, and informed consent was provided by each participant’s parent or guardian.

**Table 1:** Participants’ demographics

Subject	Age (years)	Height (m)	Mass (kg)	CP Type	GMFCS
CP01	6.50	1.13	18.00	Hemiplegic	I
CP02	11.16	1.43	30.60	Diplegic	II
CP03	7.39	1.19	21.30	Diplegic	I
TD01	10.45	1.38	32.90	-	-
TD02	6.55	1.17	21.30	-	-
TD03	6.96	1.16	19.00	-	-

*CP*–cerebral palsy; *m*–metre; *kg*–kilogram; *TD*–typically developing; GMFCS–gross motor function classification scale

**Table 2:** List of the muscle tendon units included in the neuromusculoskeletal model

	Musculotendon units		Musculotendon units
1	Adductor brevis	18	Psoas
2	Adductor longus	19	Peroneus longus*
3	Adductor magnus1	20	Peroneus brevis
4	Adductor magnus2	21	Peroneus tertius
5	Adductor magnus3	22	Lateral gastrocnemius*
6	Biceps femoris long*	23	Medial gastrocnemius*
7	Biceps femoris short	24	Soleus*
8	Gluteus maximus1	25	Tibialis anterior*
9	Gluteus maximus2	26	Semimembranosus*
10	Gluteus maximus3	27	Semitendinosus
11	Gluteus medius1	28	Sartorius*
12	Gluteus medius2	29	Vastus lateralis*
13	Gluteus medius3	30	Vastus medialis*
14	Gluteus minimus1	31	Vastus intermedius
15	Gluteus minimus2	32	Rectus femoris*
16	Gluteus minimus3	33	Tensor fasciae latae*
17	Iliacus	34	Gracilis*

^*^indicates muscles for which experimental EMG data were available

### Data processing

Motion capture data were cleaned and labelled in Vicon Nexus 2.6, then processed in MATLAB using the MOtoNMS toolbox (Mantoan et al. 2015). For all gait cycles, henceforth referred to as trials, both marker trajectories and ground reaction force data were filtered using 4th order 6 Hz low-pass Butterworth zero-lag filter. Bipolar recorded EMG signals, from 13 selected muscles (Table 2) of the lower limb, were band-pass filtered (zero-lag 4th order Butterworth, 30–400 Hz), full-wave rectified, low-pass filtered (zero-lag 4th order Butterworth, 6 Hz) and then normalised to each muscle’s maximal excitation identified across all walking trials (Devaprakash et al. 2016), which produced the muscle excitations. Finally, the electromechanical delay was set to 50 ms (Savage et al. 2023).

### NMSK model scaling and parameter tuning

Each participant’s model was based on the OpenSim gait2392 generic model (Delp et al. 1990). To match each participant’s size, the generic musculoskeletal model was linearly scaled using motion capture data (Kainz et al. 2017), where individual bone scaling factors were calculated by minimising the Euclidean distances between corresponding experimental and virtual markers. MTU pathways defined by muscle origin, insertion, and via points were also scaled with the attached bones. Joint angles, joint moments, and MTU kinematics were respectively calculated using the inverse kinematics, inverse dynamics, and muscle analysis tools in OpenSim (v 3.3) (Delp et al. 2007). MTU parameters were tuned by morphometric scaling, wherein optimal fibre length and tendon slack lengths were optimised to ensure muscle fibres operated in the same region of the force–length and force–velocity curves as what established in the unscaled generic model (Modenese et al. 2016). The generic maximal isometric force value of each MTU was scaled based on each participant’s mass (Krogt et al. 2016) as:

1 $${F}_{s}^{{\text{max}}}={F}_{iso}^{{\text{max}}}{\left(\frac{{m}_{s}}{{m}_{{\text{g}}}}\right)}^{2/3}$$

where ${m}_{s}$ is the mass of the participant, while ${m}_{g}$ and ${F}_{{\text{iso}}}^{{\text{max}}}$ are the mass and maximal isometric force values from the unscaled template model.

MTU kinematics and inverse dynamics calculated from the complete OpenSim musculoskeletal model, as well as experimental muscle excitations, were used as input to 34 MTU’s (Table 2) (Sartori et al. 2012) and to four degrees of freedom (i.e. hip flexion/extension and adduction/abduction, knee flexion/extension, and ankle plantar/dorsi-flexion) of the instrumented leg within the calibrated EMG-informed NMSK modelling (CEINMS) toolbox (Pizzolato et al. 2015).

### NMSK model calibration and execution

CEINMS was used in a two-step process: Calibration and execution of each person’s NMSK model. Muscle activations were determined from muscle excitations using a nonlinear second-order activation dynamic model (Lloyd and Besier 2003). Muscle forces were then calculated from muscle activation and muscle–tendon kinematics using a modified Hill-type MTU model, which incorporated a muscle contractile element and a parallel elastic component in series with an elastic tendon (Hill 1938; Lloyd and Besier 2003; Pizzolato et al. 2015). In this study, independent of the approach evaluated (i.e. EMG-assisted, static optimisation or synergy-informed), all models and simulations had the same Hill-type MTU model, which included an elastic tendon and a passive parallel elastic component. For all evaluated approaches, the calibrated MTU parameters (see below) were used.

An established calibration process in CEINMS was used to adjust the parameters that govern the muscle activation and MTU dynamics to the individual (Sartori et al. 2012; Pizzolato et al. 2015; Bennett et al. 2022). The calibration was performed using four of 14 processed trials for each participant. The remaining ten trials were used to evaluate the performance of the developed workflow. In the calibration, the MTU parameters were allowed to vary to minimise experimental joint moment tracking errors (Sartori et al. 2014; Pizzolato et al. 2015; Hoang et al. 2018). Specifically, the initial optimal fibre lengths in children with CP were reduced by 0.7 to represent the effect of overstretched sarcomeres during muscle contractions (Mathewson and Lieber 2015), and then were allowed to vary by ± 10% from their initial values while tendon slack lengths were allowed to increase 0 to 10% to account for individual differences in both CP and TD cohorts (Barber et al. 2012). Strength factors were assigned to functional muscle groups to further tune the force producing capability of each muscle (Sartori et al. 2012) and allowed to vary between 0.5 and 1.5. Finally, muscle activation dynamics parameters were calibrated globally as: Shape factor was bounded between −3 and 0 and recursive coefficients between −1 and 0 (Pizzolato et al. 2015).

After calibration, the NMSK models were executed using two different approaches (in CEINMS) to estimate muscle forces and, subsequently joint moments: EMG-assisted and static optimisation methods. EMG-assisted modelling (Sartori et al. 2014; Pizzolato et al. 2015) uses optimisation methods to improve joint moments estimation by minimally adjusting the experimental (EMG-derived or mapped) muscle excitations and synthesising excitations of unmeasured muscles (Sartori et al. 2014). In this optimisation, the following objective function (${f}_{{\text{EMG}}-{\text{assisted}}}$) is minimised:

2 $$f_{{\text{EMG - assisted}}} = \alpha E_{{{\text{moment}}}} + \beta E_{{{\text{sumExc}}}} + \gamma E_{{{\text{EMG}}}}$$

where ${E}_{{\text{sumExc}}}$ are the sum squared excitations, ${E}_{{\text{moment}}}$ the joint moments tracking error (between OpenSim’s inverse dynamics and CEINMS predicted joint moments), and ${E}_{{\text{EMG}}}$ the muscle excitations tracking error (between experimental and adjusted muscle excitations). $\alpha$, $\beta$ and $\gamma$ are positive weighting coefficients. In this study, $\alpha$ and $\beta$ were set to 1, and $\gamma$ was optimised to balance between ${E}_{{\text{moment}}}$ and ${E}_{{\text{EMG}}}$ (Sartori et al. 2014). For static optimisation, *α* and *β* were set to 1 and *γ* set to 0.

**Fig. 1:**
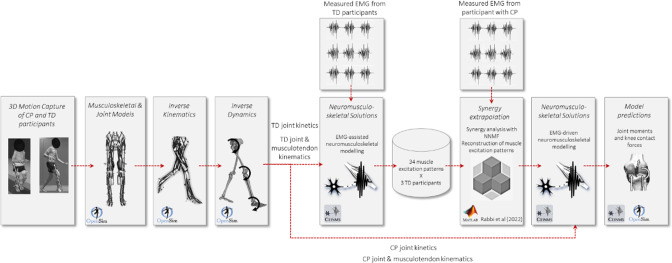
Workflow of the synergy-informed neuromusculoskeletal modelling. Motion capture data were used to create the musculoskeletal and joint models of each participant. Inverse kinematics, inverse dynamics, and muscle analysis tools in OpenSim (Delp et al., 2007) were used to calculate the joint kinetics and MTU kinematics in each participant. CEINMS (Pizzolato et al. 2015) was used to run two NMSK solutions: EMG-assisted and EMG-driven. First, EMG-assisted approach with 13 experimental muscle excitations were used to generate full set of 34 muscle excitations for all the TD participants. Then, using a small set of *m* experimental muscle excitations from each individual participant (CP and TD), their remaining (34 − *m*) muscle excitations were estimated using synergy extrapolation (Rabbi et al. 2022). Finally, EMG-driven NMSK solution was used with full set of extrapolated muscle excitations to estimate joint moments and knee contact forces in the participant with CP

The EMG-assisted approach was used to create a set of lower limb muscle excitations for all 34 muscles for each TD participant (Fig. 1). Specifically, each excitation set was created by combining the 13 experimentally EMG-derived muscle excitations and 21 muscle excitations synthesised via the EMG-assisted approach. Each excitation set were then assembled into the complete TD excitations dataset, which was then used for the synergy-informed modelling to estimate unmeasured muscle excitations in both CP and TD cohorts.

### Synergy-informed NMSK modelling

The proposed synergy-informed NMSK modelling workflow (Fig. 1) combined our synergy extrapolation method (Rabbi et al. 2022) with an EMG-driven NMSK model. The goals of the synergy-informed approach were (i) estimating muscle forces and joint contact forces from a small set of experimental muscle excitations, and (ii) ensuring that the extrapolated muscle excitations produced muscle forces that were consistent with the joint moments calculated from inverse dynamics.

The synergy extrapolation method must identify both (i) synergy excitation primitives, and (ii) synergy weights from a set of muscle excitations. A muscle synergy weight matrix for all muscles (will be termed as full synergy weight matrix) was required to estimate a full set of dynamically consistent synergy excitation primitives. To this end, a full set of dynamically consistent muscle excitations, generated through the previously described EMG-assisted method (Sect. 2.4 NMSK model calibration and execution), was used for the synergy analysis. The set consisted of 34 muscle excitations for each individual in the TD cohort. For each participant and trial, a 34 × (14 × 100) [muscles × (trials × time frames)] concatenated trial data matrix was created, all trial data matrices from all TD participants were then concatenated to create the TD full muscle excitations data matrix from which muscle synergies could be generated.

For each individual (CP or TD) and trial, selected subsets of experimental excitations from *m* muscles from all trials were used to create the individual’s data matrices (${\mathbf{X}}_{{\text{m}}}^{{\text{Ind}}}$). Then, non-negative matrix factorisation (Rabbi et al. 2020) was used to extract a set of individual muscle synergy weights (${{\varvec{W}}}_{{\text{sm}}}^{{\text{Ind}}}$) and excitation primitives (${{\varvec{H}}}_{{\text{sm}}}^{{\text{Ind}}}$) matrices for *s* synergies from *m* muscles from each ${{\varvec{X}}}_{{\text{m}}}^{{\text{Ind}}}$. The individual’s excitation primitives (${{\varvec{H}}}_{{\text{sm}}}^{{\text{Ind}}}$) matrices were combined with TD full muscle excitations data matrix (${{\varvec{X}}}^{{\text{TD}}}$) to estimate the individual’s full synergy weight matrix (${{{\varvec{W}}}_{{\text{Full}}}^{{\text{Ind}}}}_{{\text{sm}}}$), for a full set of muscles, using least squares as:

3 $${{{\varvec{W}}}_{{\text{Full}}}^{{\text{Ind}}}}_{{\text{sm}}}={{{{\varvec{H}}}_{{\text{sm}}}^{{\text{Ind}}}}^{+}{\varvec{X}}}^{{\text{TD}}}={\left[{{{\varvec{H}}}_{{\text{sm}}}^{{\text{Ind}}}}^{{\text{T}}}{{\varvec{H}}}_{{\text{sm}}}^{{\text{Ind}}}\right]}^{-1}{{{\varvec{H}}}_{{\text{sm}}}^{{\text{Ind}}}{\varvec{X}}}^{{\text{TD}}}$$

where $+$ represents Moore–Penrose pseudoinverse. The next step estimated each individual’s full set of 34 muscle excitations ($\tilde{M}_{{{\text{Full}}_{{{\text{sm}}}} }}^{{{\text{Ind}}}}$) by multiplying the full synergy weights matrix (${{{\varvec{W}}}_{{\text{Full}}}^{{\text{Ind}}}}_{{\text{sm}}}$, for* s* synergies from *m* muscles) with the individual’s excitations primitives (${{\varvec{H}}}_{{\text{sm}}}^{{\text{Ind}}}$, for* s* synergies from *m* muscles), i.e.

4 $$\tilde{\user2{X}}_{{{\text{Full}}_{{{\text{sm}}}} }}^{{{\text{Ind}}}} = {\varvec{W}}_{{{\text{Full}}_{{{\text{sm}}}} }}^{{{\text{Ind}}}} {\varvec{H}}_{{{\text{sm}}}}^{{{\text{Ind}}}}$$

In $\tilde{\user2{X}}_{{{\text{Full}}_{{{\text{sm}}}} }}^{{{\text{Ind}}}}$, the *m* estimated muscle excitations were replaced by original *m* measured excitations.

This realised *m* measured plus (34—*m*) estimated muscle excitations for each person and trial that were then used as inputs to an EMG-driven NMSK model in CEINMS to estimate muscle forces and joint moments (hip flexion/extension, hip adduction/abduction, knee flexion/extension, and ankle plantar/dorsi-flexion moments). The only difference between TD and CP groups was for each TD participant the TD full excitation data matrix included a complete set of 34 muscle excitations from the other two TD participants, estimated via the EMG-assisted approach, whereas the CP analyses used a TD data matrix constructed from the muscle excitations of all three TD participants.

### Knee joint contact model

Muscle forces and joint moments calculated from each of the NMSK models were used to estimate the knee joint contact force. Medial (^MC^) and lateral (^LC^) knee contact forces (${\text{KCF}}$) were calculated by solving the static equilibrium problem (Winby et al. 2009) as:

5 $${{\text{KCF}}}^{{\text{LC}}/{\text{MC}}}=\frac{{M}_{{\text{MTU}}}^{{\text{MC}}/{\text{LC}}}-{M}_{{\text{ext}}}^{{\text{MC}}/{\text{LC}}}}{{d}_{IC}}$$

where ${M}_{{\text{MTU}}}^{{\text{MC}}/{\text{LC}}}$ is the overall muscle moment acting on the medial/lateral knee compartment, ${M}_{{\text{ext}}}^{{\text{MC}}/{\text{LC}}}$ is the external moment around the medial/lateral contact point calculated in OpenSim, and ${d}_{IC}$ is the intercondylar distance (i.e. distance between two contact points) measured in OpenSim. Muscle moments were calculated as:

6 $${M}_{{\text{MTU}}}^{{\text{MC}}/{\text{LC}}}= \sum_{i=0}^{n}{F}_{{\text{MTU}}}^{i}{r}_{{\text{MTU}}}^{i}$$

where ${F}_{{\text{MTU}}}^{i}$ is the force generated by the *i*th MTU, and ${{\text{r}}}_{{\text{MTU}}}^{i}$ is the moment arm of *i*th MTU at the medial/lateral contact points.

### Comparing synergy-informed, EMG-informed, and static optimisation NMSK modelling predictions

For all individuals (CP and TD), we examined various combinations of *m* recorded muscle excitations and *s* synergies to estimate other (34—*m*) muscle excitations by applying synergy-informed modelling. A range of different combinations of *m* muscles and *s* synergies were piloted (Supplementary Table T1) from which four final combinations (Table 3) were selected for full evaluation. In addition, a set of 13 measured muscle excitations with 6 synergies were evaluated as another synergy-informed NMSK method to estimate muscle excitations, joint moments, and knee contact forces. These outputs were compared with corresponding estimates from the EMG-assisted and static optimisation NMSK approaches.

**Table 3:** Different combinations of *m*–muscles and *s*–synergies in the full evaluation of the synergy-informed NMSK modelling

Combination	*m* Muscles	*s* Synergies
1	13 muscles, i.e. all measured TD excitations	6
2	3 muscles (SOL, SM, VL)	3
3	4 muscles (SOL, SM, VM, VL)	3
4	4 muscles (SOL, TA, SM, VL)	4

*SOL*–soleus; *SM*–semimembranosus; *TA*–tibialis anterior; *VL*–vastus lateralis; *VM*–vastus medialis

For the models’ comparisons, the lower limb joint moments and knee contact forces were amplitude normalised to each participant’s body weight (BW). Root-mean-squared errors (RMSE) and coefficient of determination (*R*^2^) between models’ estimated joint moments and muscle excitations and the corresponding inverse dynamics joint moments from OpenSim and experimental muscle excitations were calculated to compare estimation performance of the EMG-assisted, static optimisation, and synergy-informed NMSK methods (Table 4). Values were reported as mean ± standard deviation across participant groups. Finally, the output of the EMG-assisted model, which used 13 measured muscle excitations as input, was used as reference to assess the ability of static optimisation and synergy-informed methods to estimate medial, lateral, and total knee contact forces.

**Table 4:** Performance metrics to compare EMG-assisted, static optimisation, and synergy-informed modelling approaches in both CP and TD population

Validation techniques	Quantities	Metrics
Estimation of experimental data	i. Muscle excitations ii. Joint moments iii. Knee contact forces	R^2^, RMSE
PDF similarity to experimental data	i. Muscle excitations	KS test (occurrence of agreement and maximum dissimilarity)
Information criteria	i. Goodness-of-fit ii. Number of internal variables of NMSK model	AIC, BIC

*AIC*–Akaike information criteria; *BIC*–Bayesian information criteria; *KS*–Kolmogorov–Smirnov test; *PDF*–probability density function; *RMSE*–root-mean-squared error; *R*^2^–coefficient of determination; *CP*–cerebral palsy; *TD*–typically developing

The Kolmogorov–Smirnov (KS) test was also performed to gauge the information content preserved in the estimated muscle excitations in comparison with the measured muscle excitations (Rabbi et al. 2020). In this test, the probability density function (PDF) was calculated and compared for both the measured and estimated muscle excitations to evaluate the information content estimated by the model. Furthermore, the information criteria retained by the three modelling approaches was assessed by applying the Akaike information criterion (AIC) and Bayesian information criterion (BIC) (see next section) to different sets of estimated and measured muscle excitations to examine the better modelling approach when using the least number of EMG recordings (Table 4).

### Information criteria applied to NMSK modelling

To determine the most appropriate modelling approach, we evaluated three models (i.e. EMG-assisted, static optimisation, and synergy-informed, Table 4) as a function of the trade-off between the number of model’s internal variables and the goodness-of-fit of the model’s outputs. Specifically, with a set of input observations (i.e. measured muscle excitations, inverse kinematics, and inverse dynamics) each modelling approach required to calculate different number of internal variables (e.g. unmeasured muscle excitations and four joint moments) with different accuracy. We assessed which modelling approach best estimated both muscle excitations and joint moments while requiring the least amount of information.

To-this-end, the Akaike information criterion (AIC) and Bayesian information criterion (BIC) (Akaike 1974; Schwarz 1978) were used to determine which modelling approach worked best with the least amount of experimental EMG data. The AIC and BIC were calculated as:

7 $${\text{AIC}}=2k-2{\text{ln}}\left(\widehat{L}\right)$$

8 $${\text{BIC}}=k{\text{ln}}\left(n\right)-2{\text{ln}}\left(\widehat{L}\right)$$

where $n$ is the number of input observations and *k* represents the number of internal variables within the model. $\widehat{L}=p\left(x | \widehat{\theta };M\right)$ is the probability of observing $x$ given the best matched parameter, $\widehat{\theta }$ in a model, $M$. In other words, $\widehat{L}$ is the maximised value of the likelihood function being the measure of goodness-of-fit of the modelling method. Consequently, the minimum AIC and BIC values would indicate the best performing model.

To determine the number of internal variables (*k*), we need to calculate the number of input observation (*n*) which is obtained from independent database of three TD children. For each participant with *m* experimental muscle excitations, *n* was calculated from the number of time points in joint angles (100 × 4), joint moments (100 × 4), MTU lengths (100 × 34), excitations of *m* measured muscles as, *n* = 100 × 4 + 100 × 4 + 100 × 34 + 100 × *m.* Similarly for each participant, *k* was calculated from the number of time points (i.e. 100), the number of estimated muscles (34 – *m*), estimated joint moments (100 × 4), and/or number of synergy excitation primitives (*s*) and number of synergy weights (*s*) depending on the type of model:

$$ \begin{aligned} k_{{\text{EMG - assisted}}} = & \;\;{1}00 \, \times \, \left( {{34 }{-}m} \right) \, + { 1}00 \, \times {\text{ 4 for EMG}} - {\text{assisted}}\;{\text{modelling}}, \\ k_{{{\text{static\_optimisation}}}} = & \;\;{1}00 \, \times { 34 } + { 1}00 \, \times {\text{ 4 for static}}\;{\text{optimisation}}\;{\text{modelling}},\;{\text{and}} \\ k_{{\text{synergy-informed}}} = & \;\;{1}00 \, \times s + \, \left( {{34 }{-}m} \right) \, \times s + { 1}00 \, \times {\text{ 4 for}}\;{\text{synergy}}-{\text{informed}}\;{\text{modelling}}. \\ \end{aligned} $$

Note that, all three NMSK modelling approaches used the calibrated models while information criteria (i.e. AIC and BIC) were calculated in execution stage of the CEINMS workflow. This means inverse kinematics and MTU parameters remained the same for all three approaches during the estimation of unmeasured muscle excitations and joint moments, and thereby excluded from the AIC and BIC calculations. Muscle excitations and joint moments tracking errors were assumed to be normally distributed with variance ${\sigma }_{l}^{2}$ and ${\sigma }_{j}^{2}$, respectively. A MATLAB function *aicbic()* was used to calculate AIC and BIC for all three NMSK modelling setups.

### Statistical analyses

For all models and muscle combinations, the muscle excitations and joint moments were compared based on RMSE and *R*^2^ between the experimental and estimated data. The KS test was performed to compare similarity of PDFs between experimental and estimated muscle excitations from all models using occurrence of agreement and maximum dissimilarity. Further, knee contact forces (lateral, medial, and total) estimated by the static optimisation and synergy-informed modelling were compared to those estimated by EMG-assisted modelling approach using RMSE and *R*^2^. The individual evaluation metrics for muscle excitations, joint moments, and knee contact forces were compared using repeated measures analysis of variance (ANOVA) with Bonferroni correction.

## Results

**Fig. 2:**
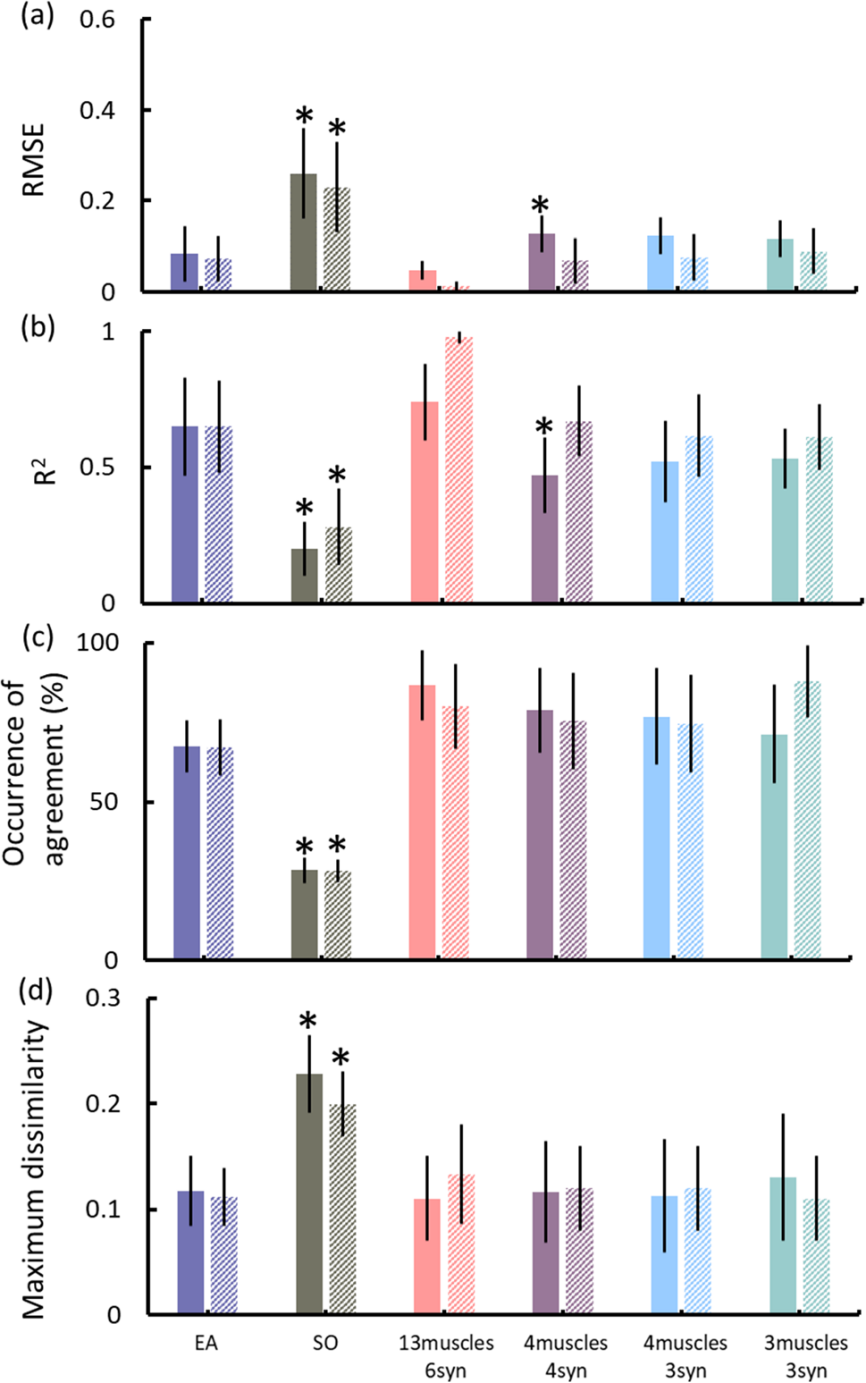
**a** RMSE and **b** *R*^2^ of the experimental muscle excitation tracking by EMG-assisted, static optimisation, and synergy-informed NMSK methods. Kolmogorov–Smirnov test results showing (**c**) occurrence of agreement and **d** maximum dissimilarity of probability density functions between the experimental and estimated muscle excitations using different model and muscle combinations. Solid and shaded bars present CP and TD participants, respectively. Error bar represents standard deviation across participants. *represents significant differences (*p* < 0.05) from the results of EMG-assisted approach; CP–cerebral palsy; EA–EMG-assisted; RMSE–root-meant-squared error; *R*^2^–coefficient of determination; SO–static optimisation; syn–synergies; TD–typically developing

Compared to the EMG-assisted method, the synergy-informed NMSK approach (Table 3) generated similar muscle excitations in terms of RMSE and *R*^2^ for both CP and TD groups, except for four muscles with four synergies (Fig. 2a, b). Importantly, the muscle excitations estimated by static optimisation were statistically different and the worst performer among evaluated models. Further, the KS test did not reveal any statistically significant difference of probability density function between the experimentally collected and reconstructed muscle excitations when either synergy-informed or EMG-assisted approach was used (Fig. 2c, d). However, KS test results from static optimisation were significantly different, and poorer, than KS results from the EMG-assisted approach. The performance of experimental muscle excitations’ tracking with synergy-informed approach using combinations of muscles different from what listed in Table 3 can be found in Supplementary Table T1. Joint angles calculated from inverse kinematics were also reported for qualitative assessment (Supplementary Figures S1 and S2).

**Fig. 3:**
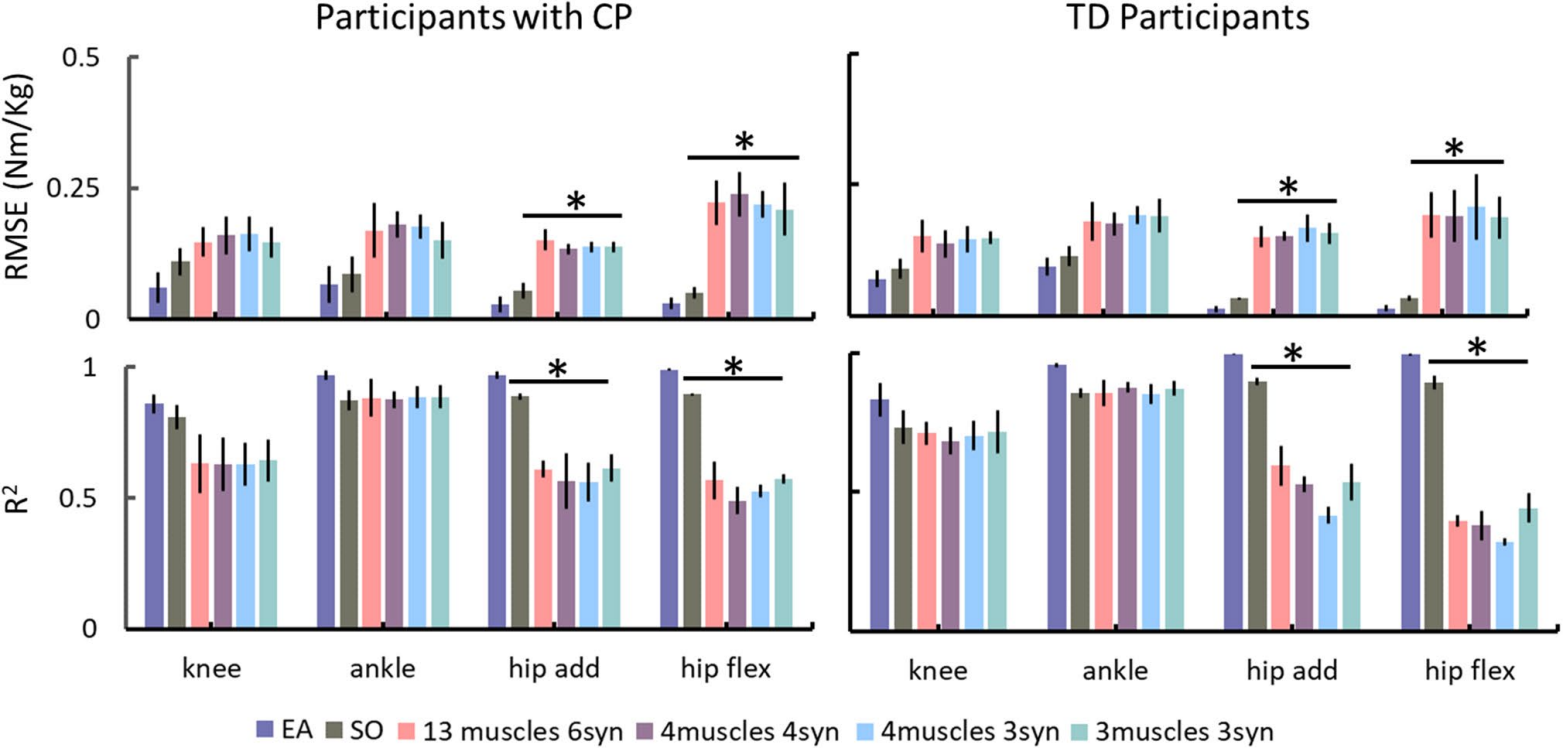
Tracking of inverse dynamics calculated joint moments using EMG-assisted, static optimisation, and synergy-informed NMSK methods in participants with CP and TD participants. Error bars represent standard deviation across participants. * indicates the significant differences (*p* < 0.05) from the results of EMG-assisted approach. Add–adduction/abduction; CP–cerebral palsy; EA–EMG-assisted; flex–flexion/extension; SO–static optimisation; TD–typically developing

Inverse dynamic’s joint moments at ankle, knee, and hip were similarly tracked by the EMG-assisted and static optimisation methods. However, EMG-assisted demonstrated superior tracking compared to the four synergy-informed NMSK models (Fig. 3 and Supplementary Figure S3). For both CP and TD cohort, *R*^2^ values were generally larger (mean difference ± 95% confidence interval values across three joints were 0.30 ± 0.13 and 0.36 ± 0.18 Nm), while RMSE were lower (0.13 ± 0.02 and 0.12 ± 0.02) than the EMG-assisted approach when compared with the same metrics in synergy-informed models. The observed differences of estimates were statistically significant at the hip, but not at the ankle and knee (Fig. 3) when compared with EMG-assisted approaches for both synergy-informed and static optimisation approaches. The performance of joint moment tracking using combinations of muscles different from what listed in Table 3 can be found in Supplementary Table T2.

**Fig. 4:**
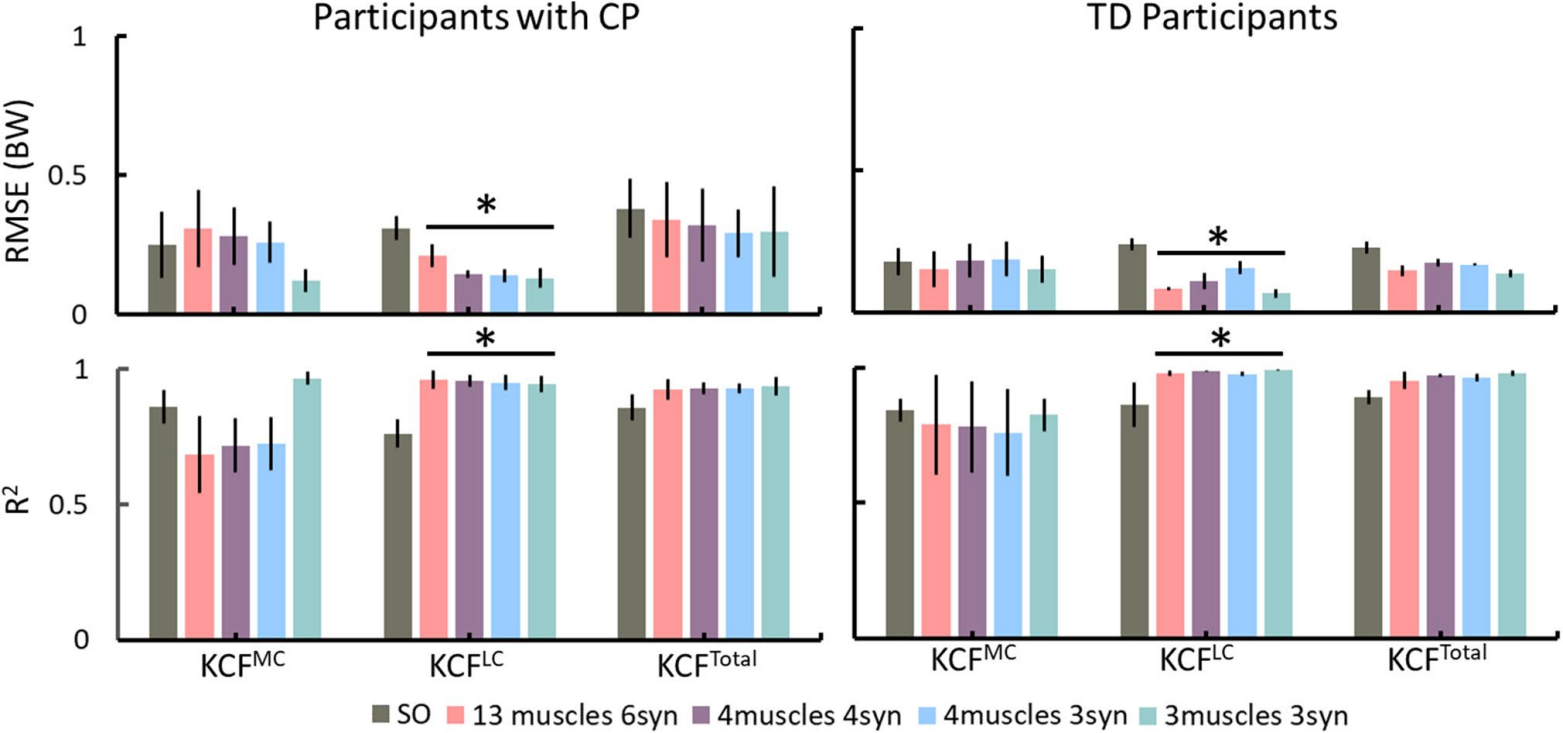
Comparison of knee contact force estimations using the static optimisation and four synergy-informed NMSK relative to the EMG-assisted models (RSME and *R*^2^). Error bars represent standard deviation across participants. *indicates the significant differences (*p* < 0.05) between static optimisation and synergy-informed approaches. CP–cerebral palsy; KCF–knee contact force; MC–medial contact; LC–lateral contact; SO–static optimisation; TD–typically developing; Total–total contact force

**Fig. 5:**
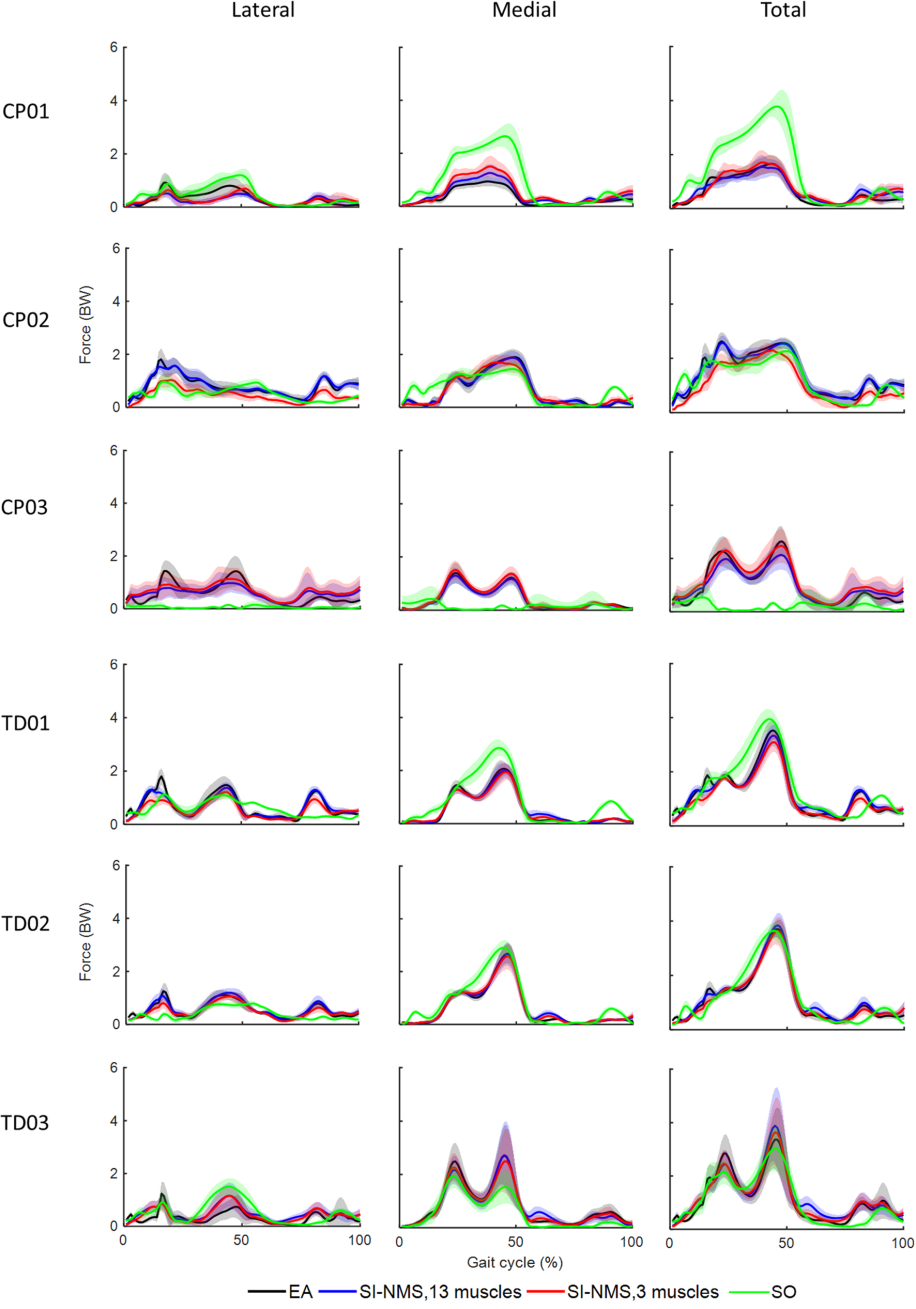
Lateral, medial, and total knee contact forces calculated with estimated joint moments using EMG-assisted (EA), and synergy-informed NMS (SI-NMS) methods, and static optimisation (SO). Solid lines and shaded regions represent the mean and standard deviation of the knee contact force across all trials

The EMG-assisted knee contact forces (KCF) were comparable (i.e. no statistical significance) between TD and CP groups by synergy-informed and static optimisation NMSK methods (Figs. 4 and 5). However, for all participants the synergy-informed approach produced estimates of lateral KCF (KCF^LC^) closer to those estimated using EMG-assisted models than those estimated using static optimisation (higher *R*^2^ and lower RMSE, *p* < 0.05), while no differences were detected with respect to the predicted medial compartment (KCF^MC^) and total (KCF^total^) loads. Considering the three or four experimental EMG recordings with three synergies, *R*^2^ (mean ± standard deviation) were 0.95 ± 0.01 and 0.93 ± 0.07 across all KCFs for CP and TD groups, respectively. Additionally, *R*^2^ from each KCFs estimated by synergy-informed method were respectively 0.66 ± 0.28, 0.96 ± 0.18, and 0.94 ± 0.11 for KCF^MC^, KCF^LC^, and KCF^total^ for the participants with CP. The performance of estimating EMG-assisted knee contact forces with synergy-informed NMSK approaches using all muscle combinations are available in Supplementary Table T3.

The information criteria analyses (Table 5) showed that both AIC and BIC calculated in synergy-informed NMSK, and static optimisation methods were significantly lower and higher (*p* < 0.05), respectively, than those calculated in EMG-assisted approach. Through maximisation of the log-likelihood function with the lowest number of internal variables, synergy-informed NMSK with three muscles, the use of three synergies was able to produce lowest AIC and BIC among all other compared models including participants from both CP and TD groups.

**Table 5:** Mean ± standard deviation AIC and BIC values for different modelling setups for both CP and TD groups

Model, *m* experimental EMG recordings, *s* synergies	# internal variables	AIC	BIC
EMG-assisted, 13 EMG, 0 synergy	2500	4723.82 ± 176.30	21,255.08 ± 176.30
Static optimisation, 0 EMG, 0 synergy	3800	7384.22 ± 172.30^*^	31,487.02 ± 176.30^*^
synergy-informed NMSK, 13 EMG, 6 synergies	1364	2583.79 ± 40.99^*^	11,599.47 ± 38.09^*^
synergy-informed NMSK, 4 EMG, 4 synergies	920	1324.58 ± 130.17^*^	7338.55 ± 113.06^*^
synergy-informed NMSK, 4 EMG, 3 synergies	790	1064.52 ± 128.20^*^	6242.15 ± 112.33^*^
synergy-informed NMSK, 3 EMG, 3 synergies	793	811.73 ± 133.96^*^	5896.32 ± 135.97^*^

^*^indicates significant differences (*p* < 0.05) with results from EMG-assisted approach. *AIC*–Akaike information criteria; *BIC*–Bayesian information criteria; *CP*–cerebral palsy; *TD*–typically developing

## Discussion

As it is impractical to acquire a large number of experimental EMG in paediatric cohorts, such as children with CP, we developed and evaluated a muscle synergy-informed NMSK modelling workflow that used a small number of experimental EMG recordings to estimate joint moments and knee contact forces. We have demonstrated that as few as three EMG recordings from the soleus, semimembranosus, and vastus lateralis muscles can be used to estimate joint moments and knee contact forces in individuals with CP that are comparable to the estimates achieved using 13 experimental EMG measurements. Upon further validation, our method could be readily translated into clinical services to inform treatment planning using sparse EMG data combined with standard gait analysis.

We showed that the large set of muscle excitations typically required to inform NMSK models of children with CP could be estimated from EMG recordings from only three (SOL, SM, VL) or four (SOL, SM, VM, VL or SOL, TA, SM, VL) muscles when employing a synergy-informed NMSK method. Further, synergy-informed NMSK modelling resulted in estimates of muscle excitations that were superior to what estimated by a static optimisation method, and with accuracy comparable to current best EMG-assisted approach (Fig. 2). The EMG channels used in input to our synergy-informed method (Table 3) were selected based on previous investigations (Rabbi et al. 2022), which identified similar group of muscles as the best candidates to obtain optimal reconstruction of extrapolated EMG data. A limited number of studies (Bianco et al. 2017) applied a muscle synergy-based method to estimate unmeasured muscle excitations, but this is the first study to demonstrate that an existing database of EMG data could be combined with a synergy-based method to inform NMSK models.

Both combinations with three and four muscles examined in this study were able to estimate joint moments using synergy-informed NMSK method with accuracy comparable to EMG-assisted approaches in both CP and TD cohorts (Fig. 3 and Supplementary Figure S3). However, the EMG-assisted approaches tracked experimental hip joint moments better (*p* < 0.05) than synergy-informed NMSK method in both groups, likely due to the lack of experimental EMG data from hip spanning muscles in our EMG database. Also, synergy-informed NMSK modelling relied on an EMG-driven approach that, unlike the employed EMG-assisted approach, did not require tracking of external joint moments. This finding might suggest that, if appropriate EMG databases were available, it might be possible to remove the need to acquire ground reaction forces.

While static optimisation and synergy-informed methods predicted similar joint moments, the synergy-informed method produced estimates of lateral knee contact forces that were closer to the reference values (i.e. EMG-assisted approach). Results also indicated that the synergy-informed method estimated muscle excitation patterns that were more physiologically plausible that static optimisation (Fig. 2a, b) and consistent with joint dynamics. As such, if a dataset were to be created by routinely collecting EMG recordings from healthy population, our proposed synergy-informed method could be used to estimate lower limb external and internal biomechanics (e.g. joint moments and contact forces) in the healthy as well as in clinical populations by simply recording experimental EMG data from three muscles (Figs. 4 and 5).

This study assessed three NMSK modelling approaches (i.e. EMG-assisted, static optimisation, and synergy-informed) with respect to the information content of the input and estimated output data. Both EMG-assisted and synergy-informed NMSK modelling approaches were able to estimate muscle excitations with comparable probability density functions (Fig. 2c, d) suggesting equivalent neural information (Miller and Childers, 2012). Also, among three NMSK modelling approaches, the synergy-informed method was able to better estimate muscle excitations while also requiring a minimal number of internal variables (Table 5). Subsequently, synergy-informed method possessed computational simplicity and information content that was better than the other two modelling approaches, a result consistent with a previous synergy-based NMSK modelling study (Bianco et al. 2017).

While the study presents promising outcomes, it is important to acknowledge its limitations. Only data from six participants were included, and data from the three TD participants were used to establish the database for the synergy extrapolation method. Critically, the limited sample size may affect the generalisability of our results; future studies should consider extending our findings by using a larger database for synergy extrapolation and evaluating model predictions in a larger cohort of children with CP across GMFCS levels. Although knee and ankle joint moments were well estimated by the synergy-informed NMSK method, hip joint moment estimation performance was significantly lower compared to EMG-assisted approach. The main reason might be the lack of EMG recordings from hip muscles in the EMG database which was used to reconstruct the unmeasured muscle excitations (Ao et al. 2020). Although we developed personalised NMSK model's some other modelling error may remain, possibly affecting estimates of muscle excitations, joint moments, and knee contact forces. However, these modelling errors and limitations would equally affect all the NMSK modelling approaches examined in this study, and thus should not affect our conclusions. Finally, direct measurement of knee contact forces was not available for this population; consequently, we selected an EMG-assisted method as benchmark for our analyses as it resulted superior to other methods in estimating in vivo measured joint contact forces (Hoang et al. 2018, 2019; Bennett et al. 2022).

## Conclusion

We developed a synergy-informed NMSK method to estimate joint moments and knee contact forces in children with CP using EMG recordings from only three (SOL, SM, VL) or four (SOL, SM, VM, VL or SOL, TA, SM, VL) muscles. While our approach showed promise, further research with a larger cohort is needed for extensive validation. Future applications of our method in clinical gait laboratories may offers a practical alternative to extensive data collection, enabling rapid and individual-specific estimations of knee contact forces.

### Supplementary Information

Below is the link to the electronic supplementary material.

Supplementary file1 (DOCX 885 KB)
