# Supplementary material for: Muscle synergy-informed neuromusculoskeletal modelling to estimate knee contact forces in children with cerebral palsy
Source: Biomech Model Mechanobiol. 2024 Mar 9;23(3):1077–90. doi: 10.1007/s10237-024-01825-7 (PMC11101562; doi:10.1007/s10237-024-01825-7)
Supplement: Supplementary file 1 — Supplementary file1 (DOCX 885 KB) [file 10237_2024_1825_MOESM1_ESM.docx]

**Supplementary document**

**Synergy extrapolation method**

For each individual (CP or TD) and trial, selected subsets of experimental excitations from *m* muscles from all trials were used to create the individual’s data matrices ($\mathbf{X}_{m}^{\mathrm{Ind}}$). Then non-negative matrix factorisation (NMF) (Rabbi et al., 2020) was used to extract a set of individual muscle synergy weights ($\mathbf{W}_{sm}^{\mathrm{Ind}}$) and excitation primitives ($\mathbf{H}_{sm}^{\mathrm{Ind}}$) matrices for *s* synergies from *m* muscles from each $\mathbf{X}_{m}^{\mathrm{Ind}}$. The individual’s excitation primitives ($\mathbf{H}_{sm}^{\mathrm{Ind}}$) matrices were combined with TD full muscle excitations data matrix ($\mathbf{X}^{\mathrm{TD}}$) to estimate the individual’s full synergy weight matrix (${\mathbf{W}_{\mathrm{Full}}^{\mathrm{Ind}}}_{sm}$), for a full set of muscles, using least squares as:

|  |  | ${\mathbf{W}_{\mathrm{Full}}^{\mathrm{Ind}}}_{sm}={{\mathbf{H}_{sm}^{\mathrm{Ind}}}^{\boldsymbol{+}}\mathbf{X}}^{\mathrm{TD}}=\left[ {\mathbf{H}_{sm}^{\mathrm{Ind}}}^{T}\mathbf{H}_{sm}^{\mathrm{Ind}} \right]^{-1}{\mathbf{H}_{sm}^{\mathrm{Ind}}\mathbf{X}}^{\mathrm{TD}}$ | (E1) |
| --- | --- | --- | --- |

where $\boldsymbol{+}$ represents Moore-Penrose pseudoinverse. The next step estimated each individual’s full set of 34 muscle excitations (${{\tilde{\mathbf{M}}}_{\mathrm{Full}}^{\mathrm{Ind}}}_{sm}$) by multiplying the full synergy weights matrix (${\mathbf{W}_{\mathrm{Full}}^{\mathrm{Ind}}}_{sm}$, for *s* synergies from *m* muscles) with the individual’s excitations primitives ($\mathbf{H}_{sm}^{\mathrm{Ind}}$, for *s* synergies from *m* muscles), i.e.,

|  |  | ${{\tilde{\mathbf{X}}}_{\mathrm{Full}}^{\mathrm{Ind}}}_{sm}={\mathbf{W}_{\mathrm{Full}}^{\mathrm{Ind}}}_{sm}\mathbf{H}_{sm}^{\mathrm{Ind}}$ | (E2) |
| --- | --- | --- | --- |

In ${{\tilde{\mathbf{X}}}_{\mathrm{Full}}^{\mathrm{Ind}}}_{sm}$, the *m* estimated muscle excitations were replaced by original *m* measured excitations.

**Table T1.** Best experimental muscle combinations and accuracy in terms of VAF, R^2^ and RMSE for estimating three to seven excluded muscles when extracting three to seven muscle synergies.

| # *experi-mental* muscles | # synergies | muscle combinations | estimation VAF (%) mean ±std | R^2^  mean ±std | RMSE  mean ±std |
| --- | --- | --- | --- | --- | --- |
| 3 | 3 | MG SM VL | 79.93 ±9.64 | 0.77±0.02 | 0.01±0.01 |
|  |  | SOL SM VL | 73.58 ±8.40 | 0.72±0.03 | 0.01±0.01 |
|  |  | MG TA VL | 72.79 ±6.61 | 0.69±0.04 | 0.01±0.01 |
| 4 | 3 | LG SM SR VM | 79.15 ±6.40 | 0.77±0.02 | 0.01±0.01 |
|  |  | SOL SM VM VL | 77.18 ±5.36 | 0.74±0.01 | 0.02±0.01 |
|  |  | SOL TA SM VM | 76.94 ±4.82 | 0.72±0.03 | 0.03±0.01 |
|  | 4 | SOL TA SM VL | 71.74 ±7.40 | 0.70±0.02 | 0.01±0.01 |
|  |  | MG TA SM VL | 71.06 ±7.55 | 0.69±0.01 | 0.03±0.01 |
|  |  | MG SOL SM VL | 70.45 ±11.94 | 0.67±0.03 | 0.03±0.02 |
| 5 | 3 | LG TA SM SR VM | 77.12 ±6.6 | 0.74±0.01 | 0.01±0.01 |
|  |  | SOL TA SM VM VL | 76.72 ±3.27 | 0.73±0.02 | 0.01±0.01 |
|  |  | LG SOL SM SR VM | 76.49 ±5.82 | 0.73±0.04 | 0.01±0.01 |
|  | 4 | SOL SM SR VM VL | 74.44 ±4.5 | 0.73±0.02 | 0.01±0.01 |
|  |  | MG LG TA SM VL | 74.42 ±4.47 | 0.74±0.04 | 0.02±0.01 |
|  |  | LG SM BF VM VL | 74.12 ±5.67 | 0.71±0.01 | 0.01±0.02 |
|  | 5 | MG SOL TA SM VL | 67.00 ±10.94 | 0.66±0.04 | 0.02±0.01 |
|  |  | MG TA SM VM VL | 66.96 ±9.47 | 0.65±0.02 | 0.02±0.01 |
|  |  | MG SOL TA BF VL | 65.71±12.00 | 0.62±0.01 | 0.01±0.02 |
| 6 | 3 | MG LG TA SM SR VM | 73.75 ±7.28 | 0.72±0.06 | 0.01±0.01 |
|  |  | LG SOL TA SM SR VM | 73.61 ±6.27 | 0.71±0.03 | 0.01±0.01 |
|  |  | LG SOL TA SM VM VL | 73.22 ±5.45 | 0.70±0.02 | 0.03±0.01 |
|  | 4 | MG LG TA SM SR VM | 74.58 ±6.57 | 0.73±0.02 | 0.03±0.01 |
|  |  | MG LG TA SM VM VL | 73.53 ±4.75 | 0.72±0.02 | 0.04±0.01 |
|  |  | MG LG TA BF VM VL | 73.34 ±8.41 | 0.72±0.01 | 0.03±0.02 |
|  | 5 | MG LG TA SM SR VM | 73.77 ±4.40 | 0.72±0.03 | 0.03±0.02 |
|  |  | LG TA SM VM VL RF | 73.06 ±3.20 | 0.71±0.04 | 0.03±0.01 |
|  |  | MG LG TA BF SR VM | 72.91 ±6.33 | 0.70±0.01 | 0.04±0.01 |
|  | 6 | MG TA SM VM VL RF | 62.86 ±10.30 | 0.60±0.02 | 0.03±0.01 |
|  |  | MG SOL TA SM VM VL | 62.65 ±9.04 | 0.61±0.02 | 0.04±0.01 |
|  |  | MG SOL SM BF VM VL | 62.17 ±11.08 | 0.61±0.01 | 0.04±0.02 |
| 7 | 3 | MG LG TA BF SR VM VL | 69.31 ±13.3 | 0.66±0.06 | 0.04±0.01 |
|  |  | LG SOL TA SM SR VM VL | 68.20 ±7.71 | 0.65±0.04 | 0.05±0.03 |
|  |  | MG LG TA SM SR VM VL | 67.18 ±10.16 | 0.62±0.01 | 0.04±0.02 |
|  | 4 | MG LG TA BF SR VM VL | 70.67 ±10.55 | 0.70±0.06 | 0.01±0.01 |
|  |  | MG LG TA SM SR VM VL | 67.40 ±7.17 | 0.64±0.03 | 0.03±0.01 |
|  |  | MG LG SOL SM SR VM VL | 67.03 ±4.08 | 0.64±0.02 | 0.04±0.01 |
|  | 5 | MG LG TA BF VM VL RF | 69.35 ±6.03 | 0.66±0.06 | 0.01±0.01 |
|  |  | LG TA SM BF VM VL RF | 68.44 ±6.78 | 0.65±0.04 | 0.02±0.01 |
|  |  | MG LG TA SM BF VM VL | 66.65 ±8.96 | 0.65±0.01 | 0.02±0.02 |
|  | 6 | MG LG SM BF SR VM VL | 71.99 ±6.3 | 0.69±0.01 | 0.03±0.01 |
|  |  | MG LG TA BF SR VM VL | 69.56±5.86 | 0.68±0.02 | 0.03±0.03 |
|  |  | MG SOL SM BF SR VM VL | 68.68 ±4.39 | 0.66±0.04 | 0.04±0.03 |
|  | 7 | MG LG TA SM BF VM VL | 60.25 ±9.49 | 0.60±0.02 | 0.02±0.01 |
|  |  | MG LG TA SM VM VL RF | 60.18 ±12.12 | 0.60±0.04 | 0.03±0.02 |
|  |  | MG LG TA SM SR VM RF | 59.46 ±10.78 | 0.58±0.04 | 0.04±0.01 |

VAF – variance accounted for; R^2^ –determination of correlation; RMSE – root means squared error;

**Inverse kinematics**


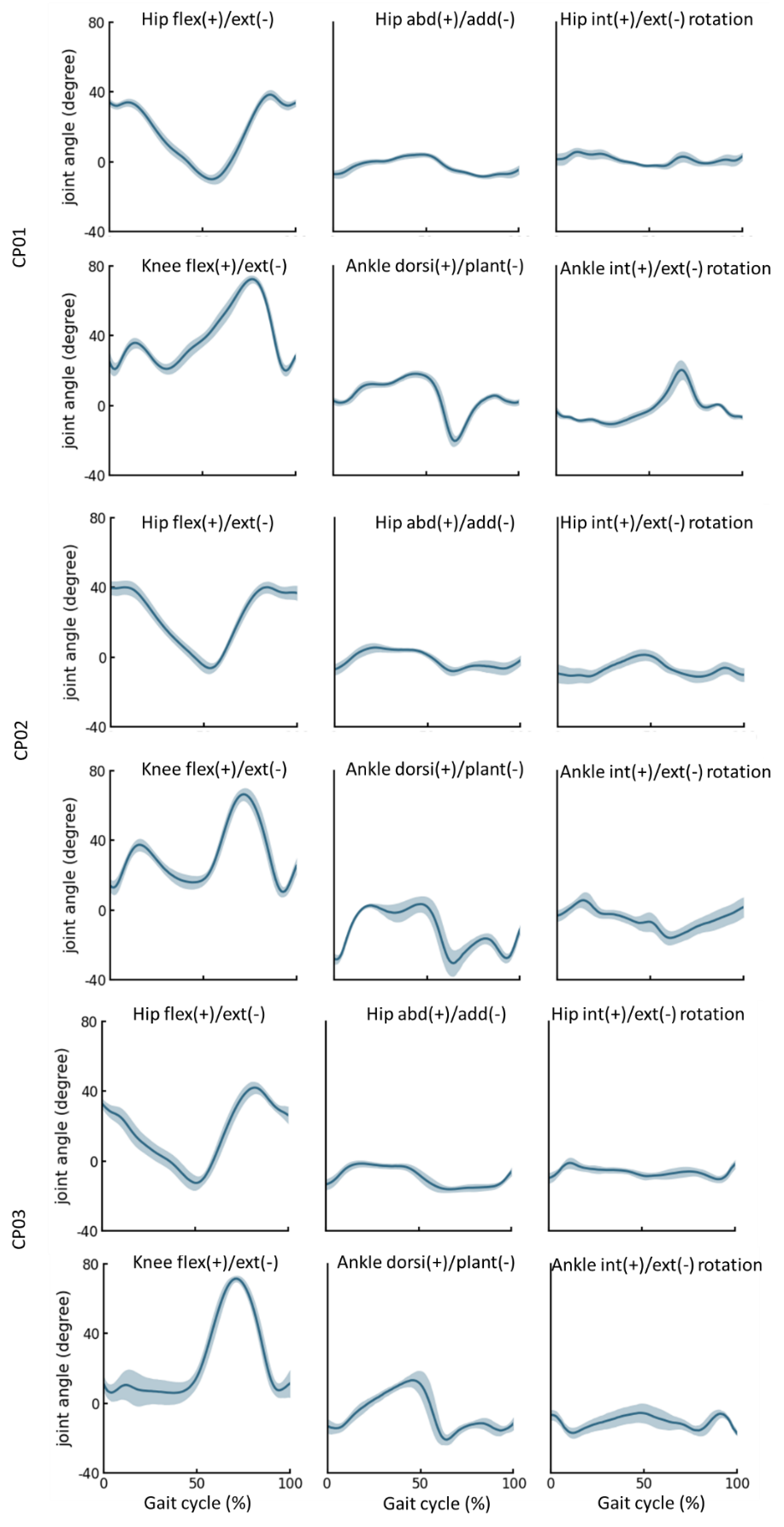


**Figure S1**. Joint angles calculated from inverse kinematics for children with CP. Solid line represents mean and shaded area represents ±1 standard deviation


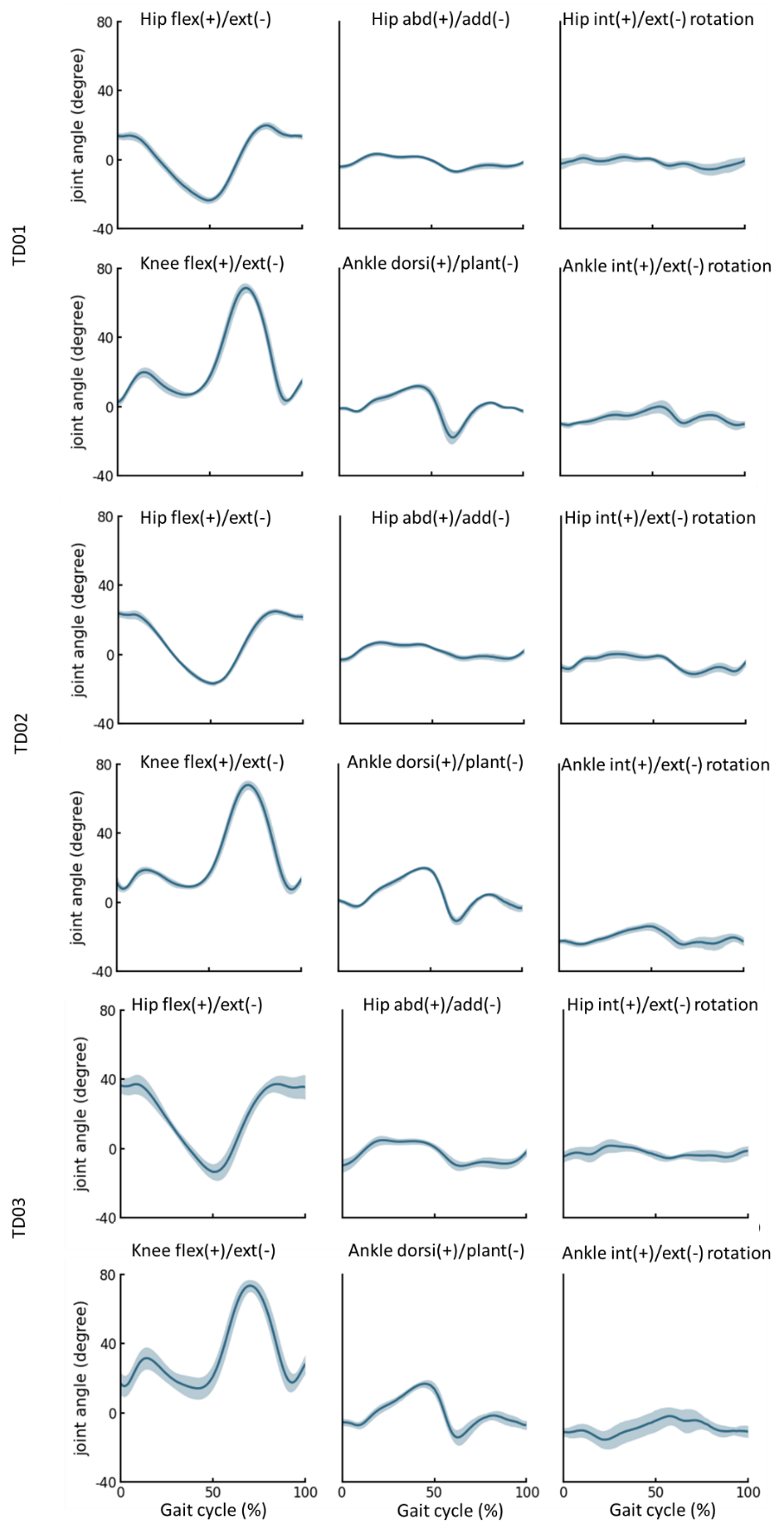


**Figure S2**. Joint angles calculated from inverse kinematics for TD children. Solid line represents mean and shaded area represents ±1 standard deviation

**Estimation of joint moments**


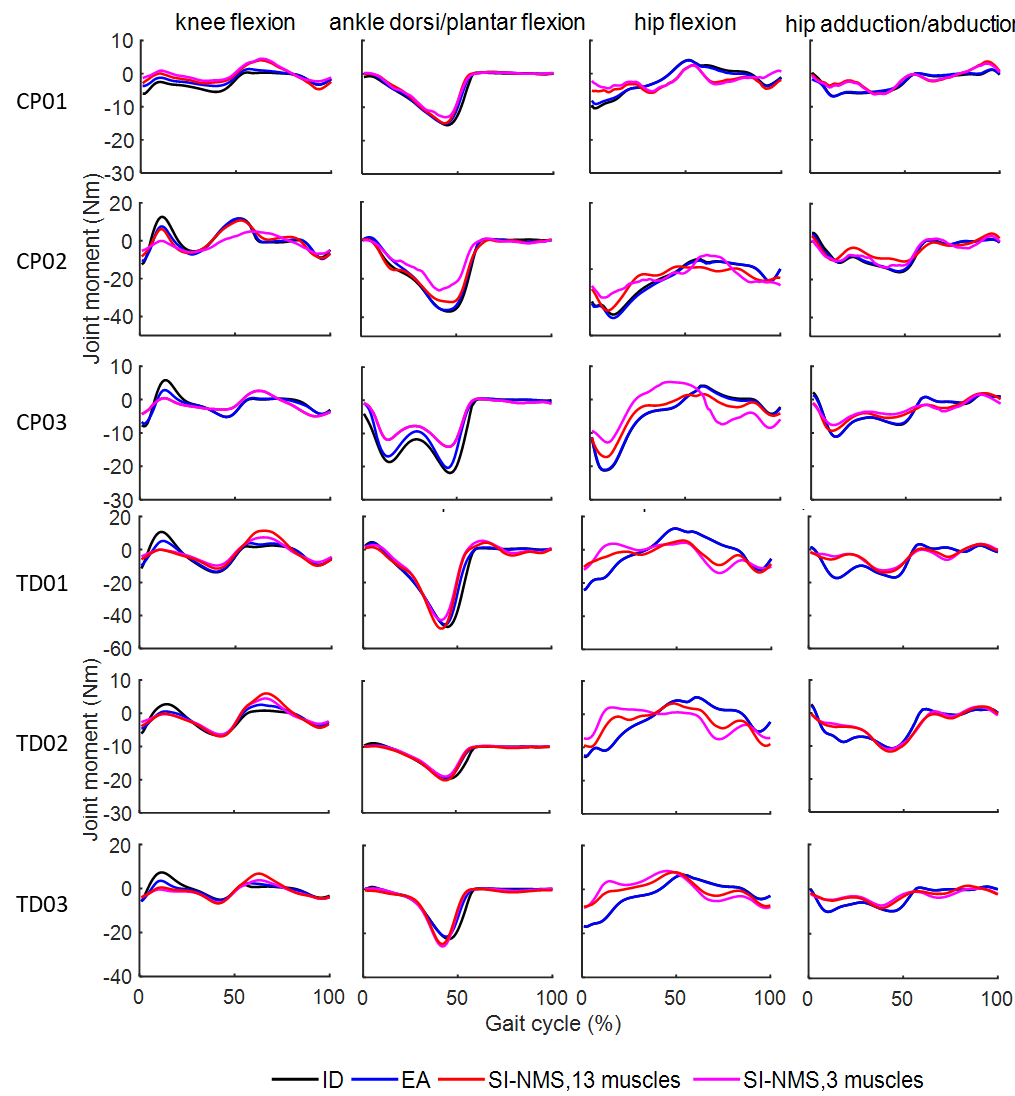


**Figure S3.** Estimated joint moments using EMG-assisted (EA) and synergy-informed NMS (SI-NMS) methods with 13 and three measured muscle excitations (SOL, SM, VL) have been compared with inverse dynamics (ID) joint moments for all participants. Solid lines represent mean joint moment across all trials.

**Table T2.** Mean ±std RMSE and R^2^ of joint moment estimation with EMG-assisted and synergy-informed NMSK methods when EMG recordings from different muscle combinations were used as the input to the model.

|  |  |  | CP | | TD | |
| --- | --- | --- | --- | --- | --- | --- |
| Model | Synergy | Moment | RMSE (Nm) | R^2^ | RMSE (Nm) | R^2^ |
| EMG assisted with 13 measured muscles | - | Knee flex | 1.41 ±0.41 | 0.90 ±0.06 | 1.68 ±0.42 | 0.83 ±0.08 |
|  |  | Ankle flex | 1.44 ±1.44 | 0.97 ±0.01 | 2.30 ±0.51 | 0.95 ±0.01 |
|  |  | Hip add | 0.28 ±0.13 | 0.98 ±0.01 | 0.06 ±0.09 | 0.99 ±0.01 |
|  |  | Hip flex | 0.37 ±0.18 | 0.99 ±0.01 | 0.09 ±0.09 | 0.99 ±0.01 |
|  |  |  |  |  |  |  |
| Static Optimisation with 13 measured muscles | - | Knee flex | 1.56 ±0.13 | 0.90 ±0.03 | 2.08 ±0.25 | 0.81 ±0.08 |
|  |  | Ankle flex | 1.66 ±1.42 | 0.92 ±0.04 | 2.10 ±0.12 | 0.90 ±0.03 |
|  |  | Hip add | 0.48 ±0.31 | 0.97 ±0.01 | 0.13 ±0.16 | 0.95 ±0.02 |
|  |  | Hip flex | 0.67 ±0.10 | 0.96 ±0.02 | 0.18 ±0.17 | 0.94 ±0.01 |
|  |  |  |  |  |  |  |
| SI-NMS  with all 13 measured muscles | 6 | Knee flex | 3.29 ±0.61 | 0.53 ±0.15 | 3.75 ±0.75 | 0.59 ±0.15 |
|  |  | Ankle flex | 3.68 ±0.70 | 0.88 ±0.03 | 4.50 ±0.78 | 0.85 ±0.04 |
|  |  | Hip add | 3.47 ±0.32 | 0.61 ±0.15 | 3.76 ±0.74 | 0.59 ±0.16 |
|  |  | Hip flex | 4.98 ±0.55 | 0.56 ±0.22 | 4.46 ±1.16 | 0.39 ±0.23 |
|  |  |  |  |  |  |  |
| SI-NMS  (MG, SM, VL) | 3 | Knee flex | 4.45 ±1.06 | 0.55 ±0.15 | 3.28 ±0.57 | 0.52 ±0.15 |
|  |  | Ankle flex | 4.06 ±1.85 | 0.88 ±0.06 | 4.55 ±0.83 | 0.87 ±0.04 |
|  |  | Hip add | 3.12 ±0.09 | 0.58 ±0.17 | 4.25 ±0.84 | 0.48 ±0.19 |
|  |  | Hip flex | 5.66 ±2.37 | 0.50 ±0.11 | 8.53 ±1.38 | 0.10 ±0.16 |
|  |  |  |  |  |  |  |
| SI-NMS  (SOL, SM, VL) | 3 | Knee flex | 3.26 ±0.94 | 0.64 ±0.12 | 3.60 ±0.44 | 0.60 ±0.09 |
|  |  | Ankle flex | 3.49 ±1.34 | 0.88 ±0.04 | 4.44 ±1.0 | 0.87 ±0.03 |
|  |  | Hip add | 3.21 ±1.01 | 0.61 ±0.21 | 3.74 ±0.93 | 0.53 ±0.18 |
|  |  | Hip flex | 4.65 ±0.84 | 0.57 ±0.12 | 4.43 ±1.2 | 0.44 ±0.07 |
|  |  |  |  |  |  |  |
| SI-NMS  (LG, SM, SR, VM) | 3 | Knee flex | 4.51±0.81 | 0.48 ±0.13 | 3.57 ±0.59 | 0.45 ±0.12 |
|  |  | Ankle flex | 4.13±1.31 | 0.88 ±0.04 | 4.76 ±0.94 | 0.85 ±0.05 |
|  |  | Hip add | 3.21±1.0 | 0.56 ±0.21 | 4.39 ±0.65 | 0.46 ±0.14 |
|  |  | Hip flex | 7.67±2.17 | 0.35 ±1.22 | 8.43 ±1.28 | 0.09 ±0.12 |
|  |  |  |  |  |  |  |
| SI-NMS  (SOL, SM, VM, VL) | 3 | Knee flex | 4.38 ±0.86 | 0.62 ±0.14 | 3.68 ±0.68 | 0.46 ±0.15 |
|  |  | Ankle flex | 4.16±1.34 | 0.88 ±0.04 | 4.66 ±0.91 | 0.85 ±0.05 |
|  |  | Hip add | 3.21±1.0 | 0.56 ±0.21 | 3.94 ±0.87 | 0.41 ±0.17 |
|  |  | Hip flex | 5.01±0.60 | 0.52 ±0.12 | 4.76 ±1.9 | 0.32 ±0.10 |
|  |  |  |  |  |  |  |
| SI-NMS  (SOL, TA, SM, VL) | 4 | Knee flex | 4.33±0.9 | 0.57 ±0.14 | 3.48 ±0.64 | 0.53 ±0.13 |
|  |  | Ankle flex | 4.16 ±1.2 | 0.87 ±0.05 | 4.39 ±0.87 | 0.87 ±0.05 |
|  |  | Hip add | 3.11 ± 0.76 | 0.56 ±0.18 | 3.71 ±0.84 | 0.52 ±0.18 |
|  |  | Hip flex | 5.34 ±0.54 | 0.48 ±0.16 | 4.38 ±0.86 | 0.37 ±0.20 |
|  |  |  |  |  |  |  |
| SI-NMS  (MG, TA, SM, VL) | 4 | Knee flex | 4.06 ±0.85 | 0.45 ±0.14 | 3.40 ±0.63 | 0.57 ±0.13 |
|  |  | Ankle flex | 4.75 ±1.2 | 0.82 ±0.07 | 5.14 ±0.67 | 0.80 ±0.05 |
|  |  | Hip add | 3.14 ±0.87 | 0.57 ±0.20 | 4.04 ±0.79 | 0.52 ±0.16 |
|  |  | Hip flex | 7.7 2±2.4 | 0.39 ±0.17 | 5.57 ±1.93 | 0.29 ±0.21 |

LG– lateral gastrocnemius; MG– medial gastrocnemius; SR– sartorius; SM– semimembranosus; SOL– soleus; std– standard deviation; TA– tibialis anterior; VL– vastus lateralis; VM– vastus medialis.

**Table T3.** Mean ±std RMSE and R^2^ of knee contact forces estimation with Synergy-informed NMS (SI-NMS) versus EMG-assisted method when different muscle combinations were used in synergy extrapolation.

|  |  |  | CP | | TD | |
| --- | --- | --- | --- | --- | --- | --- |
| Model | Synergy | JCF | RMSE | R^2^ | RMSE | R^2^ |
| SI-NMS with all 13 measured muscles | 6 | Medial | 0.30 ±0.12 | 0.68 0.14 | 0.15 ±0.06 | 0.78 ±0.18 |
|  |  | Lateral | 0.14 ±0.04 | 0.98 ±0.02 | 0.06 ±0.01 | 0.99 ±0.01 |
|  |  | Total | 0.34 ±0.13 | 0.92 ±0.03 | 0.14 ±0.01 | 0.97 ±0.03 |
| SI-NMS  (MG, SM, VL) | 3 | Medial | 0.29 ±0.10 | 0.75 ±0.07 | 0.18 ±0.04 | 0.76 ±0.05 |
|  |  | Lateral | 0.19 ±0.04 | 0.95 ±0.01 | 0.08 ±0.01 | 0.98 ±0.01 |
|  |  | Total | 0.33 ±0.13 | 0.91 ±0.01 | 0.18 ±0.04 | 0.97 ±0.01 |
| SI-NMS  (SOL SM VL) | 3 | Medial | 0.26 ±0.22 | 0.96 ±0.02 | 0.18 ±0.04 | 0.75 ±0.05 |
|  |  | Lateral | 0.12 ±0.04 | 0.76 ±0.03 | 0.10 ±0.01 | 0.99 ±0.01 |
|  |  | Total | 0.29 ±0.16 | 0.93 ±0.03 | 0.17 ±0.13 | 0.97 ±0.01 |
| SI-NMS  (LG SM SR VM ) | 3 | Medial | 0.35 ±0.16 | 0.86 ±0.14 | 0.18 ±0.04 | 0.74 ±0.12 |
|  |  | Lateral | 0.49 ±0.09 | 0.66 ±0.09 | 0.15 ±0.04 | 0.97 ±0.01 |
|  |  | Total | 0.69 ±0.20 | 0.84 ±0.08 | 0.21 ±0.03 | 0.96 ±0.01 |
| SI-NMS  (SOL SM VM VL) | 3 | Medial | 0.28 ±0.07 | 0.71 ±0.09 | 0.18 ±0.06 | 0.75 ±0.16 |
|  |  | Lateral | 0.14 ±0.02 | 0.95 ±0.03 | 0.15 ±0.04 | 0.97 ±0.01 |
|  |  | Total | 0.32 ±0.08 | 0.92 ±0.01 | 0.22 ±0.02 | 0.96 ±0.01 |
| SI-NMS  (SOL TA SM VL) | 4 | Medial | 0.28 ±0.10 | 0.71 ±0.10 | 0.18 ±0.05 | 0.77 ±0.16 |
|  |  | Lateral | 0.14 ±0.01 | 0.95 ±0.02 | 0.11 ±0.02 | 0.98 ±0.01 |
|  |  | Total | 0.32 ±0.13 | 0.92 ±0.02 | 0.19 ±0.14 | 0.96 ±0.01 |
| SI-NMS  (MG TA SM VL) | 4 | Medial | 0.31 ±0.12 | 0.58 ±0.19 | 0.17 ±0.05 | 0.76 ±0.18 |
|  |  | Lateral | 0.21 ±0.06 | 0.96 ±0.01 | 0.06 ±0.01 | 0.99 ±0.01 |
|  |  | Total | 0.35 ±0.17 | 0.88 ±0.06 | 0.18 ±0.06 | 0.96 ±0.01 |

LG– lateral gastrocnemius; MG– medial gastrocnemius; SR– sartorius; SM– semimembranosus; SOL– soleus; std– standard deviation; TA– tibialis anterior; VL– vastus lateralis; VM– vastus medialis.
